# Gene expression of angiogenesis and apoptotic factors in female BALB/c mice with breast cancer after eight weeks of aerobic training

**DOI:** 10.22038/ijbms.2021.55582.12427

**Published:** 2021-09

**Authors:** Mohammad Mahdi Rafiei, Rahele Soltani, Mohammad Reza Kordi, Reza Nouri, Abbas Ali Gaeini

**Affiliations:** 1 Department of Sport Sciences, Kish International Campus, University of Tehran, Kish, Iran; 2 Department of Exercise Physiology, Faculty of Sport Sciences, University of Tehran, Tehran, Iran

**Keywords:** Aerobic exercise, Angiogenesis, Apoptosis, Breast Cancer, Gene expression

## Abstract

**Objective(s):**

Breast cancer is the most common cancer in women, caused by a disorder in the angiogenesis and apoptosis process. Exercise can affect the process of angiogenesis and apoptosis in the tumor tissue. Thus, the aim of the present study was to investigate the changes in angiogenesis and apoptotic factors in mice with breast cancer after 8 weeks of exercise training.

**Materials and Methods:**

Sixteen females BALB/c mice (age: 3-5 weeks and weight: 17.1 ± 0.1 g) with breast cancer were randomly divided into two groups of aerobic training and control. The aerobic training included 8 weeks and 5 sessions per week of running with an intensity of 14-20 m.min-1. HIF-1α, VEGF, miR-21 and cytochrome C, Apaf-1, caspase-9, and caspase-3 gene expressions were examined by real-time PCR. Repeated measures ANOVA, Bonferroni’s *post hoc *test, and independent samples t-test were used to analyze the data (*P*<0.05).

**Results:**

The results showed that aerobic training reduced the growth of tumor volume and significantly reduced miR-21 gene expression. Aerobic training also significantly increased the gene expression of HIF-1α, cytochrome C, Apaf-1, caspase-9, and caspase-3, while changes in VEGF gene expression were not statistically significant.

**Conclusion:**

It appears that aerobic exercise training reduces tumor size and ameliorates breast cancer by reducing miR-21 gene expression, suppressing the apoptosis process, and reducing angiogenesis.

## Introduction

Breast cancer is a multi-stage disease and includes disorders in various signaling cascades (1), including inflammatory pathways, apoptosis, and angiogenesis (2). Tumor angiogenesis is a vital process in the formation of new blood vessels and plays a critical role in tumor growth, invasion, and metastasis (1). In general, tumors cannot grow more than 1 to 2 mm^3^ without the formation of new blood vessels to transport oxygen and nutrients (3), thus they require an angiogenesis process (4). Nitric oxide (NO), hypoxia-inducible factor-1a (HIF-1α), and vascular endothelial growth factor (VEGF) are involved in the angiogenesis process (5, 6). The gene expression of VEGF in tumor cells is controlled by HIF-1α, a transcription factor that is induced under hypoxic conditions (7).

On the other hand, apoptosis is a programmed cell death to maintain the physiological balance between cell death and cell growth and is determined by indicators such as caspase activity and the release of apoptotic factors (8). Recent studies have shown that apoptosis as a common mechanism exerts cytotoxic effects in cancer patients, and any genetic or epigenetic changes in cells under apoptosis can lead to the development of cancer (1, 9). The internal pathway of apoptosis, as the most important pathway for apoptosis, is associated with changes in the permeability of the mitochondrial outer membrane and the release of apoptotic factors (2, 3). Meanwhile, cytochrome C, as one of the most important factors in apoptosis, is released to the cytosol by increasing the permeability of the mitochondrial outer membrane and leads to triggering caspase reactions in the internal pathway. By binding to the apoptosis activating factor-1 (Apaf-1), cytochrome C activates procaspase-9, then caspase-9 becomes the apoptosis initiating caspase in the mitochondrial pathway. This process eventually activates caspase-3, called executive caspase. Once activated, caspases hydrolyze and decompose many of the vital cellular proteins, causing the cell to enter an irreversible stage, i.e., cell death (10, 11). Thus, researchers look at increasing apoptosis and reducing angiogenesis as an appropriate way to overcome cancer.

 In addition, recent studies have shown that microRNAs (miRs) also act as tumor suppressants or oncogenesis and play key roles in tumor cell proliferation and tumor cell apoptosis (12, 13). They play a role in regulating VEGF expression (12, 13). Laboratory studies of several types of cancer have shown that miR-21 degradation suppresses cell proliferation and tumor growth, leading to reduced metastatic invasion (14, 15). Various measures have been taken in clinical oncology, such as chemotherapy, gamma radiation, and immunotherapy. These measures are related to the activity of apoptosis signaling pathways in cancer cells (16).

Today, exercise has been considered a preventive and effective treatment by experts to maintain and improve the quality of life of patients with breast cancer (17). Recent research has shown that exercise, depending on the severity of the activity, can prevent the progression of breast cancer in rodents (18). Exercise by affecting angiogenesis stages can alter angiogenesis (19) and reduce angiogenesis in breast tumor tissue (20). By reviewing previous studies, the dual role of exercise in the process of angiogenesis in healthy people and patients can be understood. For example, performing exercise in healthy people increases the process of angiogenesis in various tissues, especially muscle tissue (21), in patients with type 2 diabetes, reduces angiogenesis in the kidney tissue (22), and in patients with breast cancer, it reduces angiogenesis in tumor tissue (20). Exercise also activates essential intracellular mediators such as apoptosis and inflammation (23).

So far, very limited research has been done on the effect of aerobic exercise on the expression of genes in the process of angiogenesis and apoptosis and microRNAs in breast cancer. Thus, the present study examined the changes in angiogenesis and apoptotic factors in mice with breast cancer after 8 weeks of exercise training. 

## Materials and Methods

***Animals***

The present study was laboratory experimental research. Sixteen BALB/c female mice (age: 3 to 5 weeks; weight 17.25 ± 0.8 g) were purchased from the Pasteur Institute. The mice were held in a controlled condition at 22 ± 3ºC under sleep and waking cycle (12 hours of light and 12 hours of darkness, (and humidity of 40 to 60 percent and were kept in polyethylene cages without any restrictions on food and water. The BALB/c mice did training for two weeks to get introduced to the training program and were then randomly divided into two groups: training group (n=8) and control group (n=8). After the groups were identified, all mice were exposed to cancer by subcutaneous injection of MC4-L2 cancer cells (obtained from institute pastor of Iran) in two groups. The ethical approval for this study was obtained from the Physical Education Research Institute, No. IR.SSRI.REC.1395.129. Also, for ethical purposes, all procedures were performed in accordance with the ethical standards of the U.K. Animals (Scientific Procedures) Act, 1986, and associated guidelines. 

***Cell culture***

Breast duct carcinoma cells of the positive estrogen receptor (MC4-L2) were cultured in a T75 flux in DMEM/F-12 medium with 15 mM 4-(2-hydroxyethyl)-1-piperazineethanesulfonic acid (HEPES) buffer, penicillin 100 μg.ml^-1^, glutamine, streptomycin 100 μg.ml^-1,^ and 10% phosphate buffered saline (PBS). 

The supernatant was removed after filling 90% of the flux surface with the cells, and after washing with PBS, 25% of the cell surface of the plate was removed with trypsin enzyme (aminsan), and after neutralizing the enzyme with 10% PBS medium, all the content of the flux was discharged into Falcon tube and was centrifuged for three to five min at 1200 rpm; then the supernatant was removed and dissolved in a 10% PBS medium.

Finally, trypan blue (Sigma Aldrich) and Hemacytometric lam were used to determine the survival and cell count. After cell culture, the cell suspension was prepared at a density of 10 million per 1 ml of PBS buffer. 

***Tumor formation and assessment of tumor volume***

Mice were anesthetized by ketamine (100 mg.kg^-1^) and xylazine (darodar.ir) (10 mg.kg^-1^), and one million cells were injected subcutaneously into the upper right thigh area.

Counting after 10 to 14 days, the tumor was palpable at the injection site and was confirmed by laboratory methods and doctor’s diagnosis through observing the pathological image of the tumor.

After the onset of the tumor, the largest dimension of the tumor was measured each week as the length (L) and the other dimension as the width (W) with a digital caliper. To calculate tumor volume, Jones *et al*.’s formula (V = π / 6 (L2 × W)) was used (24).

***Training protocol***

The aerobic training program included running on a treadmill for eight weeks and five sessions per week. The training began in the first week at a speed of 14 m.min^-1^ for 30 min eventually increased by 20 m.min^-1^ for 30 min in the last two weeks. 

Since in aerobic training on the treadmill, the intensity and duration of the training are easily controlled by the researcher, in the present study, the training model was used (25). Details of each training session are presented in Table 1. The control group performed no exercise and continued their normal life having the tumor.

***Sampling***

Forty-eight hours after the last training session, mice in both groups were sacrificed after anesthetizing with ketamine (100 mg.kg^-1^) and xylazine (10 mg.kg^-1^). Then, tumor tissue was separated, and the necrosis portion was removed and the supernatant tumor was immediately frozen in liquid nitrogen and stored at -70°C. Tumor volume was measured *in vivo *by external caliper, Micro-computed tomography (microCT), and Positron emission tomography (PET) with 2-deoxy-2-[fluorine-18]fluoro-D-glucose (^18^F-FDG). Intra-observer reproducibility of the microCT and caliper methods were assessed by acquiring 10 repeated volume measurements. To measure inter-observer variation, volumes of a group of tumors were determined independently by two observers. 

In the laboratory, 50-100 mg of tumor tissue with 1 ml trizol (Anacell) was poured into a manual homogeneous tube, and the tissue was homogeneous. The supernatant suspension was then transferred to 1.5 ml micro-tubes for RNA extraction. The micro-tubes were centrifuged at 1500 g for 10 min at 4°C to precipitate the larger components.

***Gene expression by real time PCR***

To examine HIF-1α, VEGF, miR-21, cytochrome C, Apaf-1, caspase-9, and caspase-3 gene expression in each group, tissue analysis was performed using real-time polymerase chain reaction (real-time PCR) technique. The total RNA extraction steps were taken strictly on the basis of the trizol application, except that for extraction of miR-21 after adding trizol, incubation was performed at -70°C for one day. It was stored at – 20°C for one day, and then the subsequent extraction steps were taken respectively. The Stratagene kit was used to make HIF-1α, VEGF, miR-21, cytochrome C, Apaf-1, caspase-9, and caspase-3 cDNA. 

At the beginning of the experiment, the optimal concentration of cDNA, as well as the primers associated with each gene, were determined separately by using a serial concentration test. 

The real-time PCR program was performed on a Corbett machine for a HIF-1α, VEGF, cytochrome C, Apaf-1, caspase-9, and caspase-3 genes at 95°C for 5 min, then 45 cycles of 95°C for 20 sec and 60°C for 1 min; and for miR-21, it contained 95°C for 10 min and 45 cycles of 95°C for 10 sec, 60°C for 15 sec, and 72°C for 20 sec. Glyceraldehyde-3-phosphate dehydrogenase (GAPDH) (Creative Enzymes company) was used as the control gene of HIF-1α, VEGF, cytochrome C, Apaf-1, caspase-9, caspase-3, and U6 as the miR-21 control gene. Primers specific are presented in Table 2.

***Statistical methods***

Data were analyzed by SPSS software version 16. At first, data normalization was confirmed by the Kolmogorov-Smirnov test. To analyze tumor growth changes, repeated measures ANOVA and Bonferroni’s *post hoc* test were used, and to analyze miR-21, HIF-1α, VEGF, cytochrome C, Apaf-1, caspase-9, and caspase-3 changes of gene expression, independent samples t-test was used. The significance level was considered as *P*<0.05.

## Results

**Figure 1.**
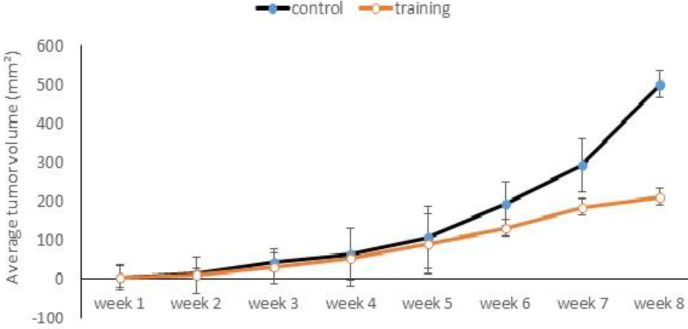
Average tumor volume changes during 8 weeks of training in both groups

The mean and standard deviation of the bodyweight of the rats are presented in Table 3. The results of repeated measures ANOVA showed that there is a significant difference between tumor volume growth in two groups (F_1, 138_=3.68, *P*=0.028). The average tumor volume in training and control groups during the eight-week aerobic training protocol is shown in Figure 1. As shown in Figure 1, the primary tumor volume in the training and control groups was approximately equal, but the final tumor growth rate in the control group was higher than the training group, and this different wasmore significant at end of training.

**Figure 2:**
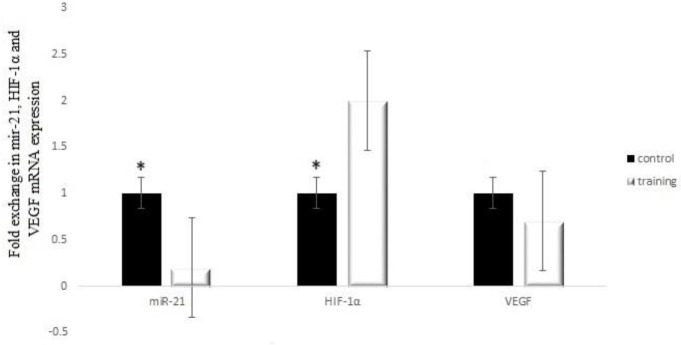
miR-21, HIF-1α, and VEGF mRNA expression in training and control groups

The results of the independent t-test for miR-21 (t_14_=1.84, *P*=0.034) and HIF-1α (t_14_=6.32, *P*=0.001) gene expression showed that there is a significant difference between the two groups (Figure 2). Also, VEGF gene expression showed no significant difference between the two groups (t_14_=1.72, *P*=0.087). As shown in Figure 2, in the training group (0.71 ± 0.13), there was no significant decrease in VEGF gene expression compared to the control group (1.01±0.12). 

**Figure 3:**
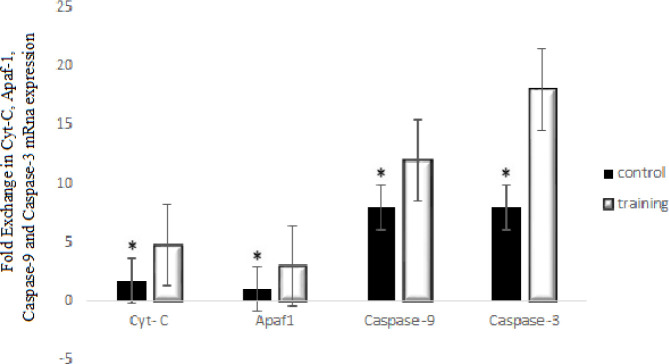
Cytochrome C, Apaf1, caspase-9, and caspase-3 mRNA expression in training and control

The results of the independent t-test for Apaf-1 gene expression showed (Figure 3) that there is a significant difference between the two groups (*t*_14_=4.55, *P*=0.001). After eight weeks of aerobic training, statistical analyses for cytochrome C (Figure 3) revealed a significant difference between two groups of control and aerobic training in the cytochrome C gene expression (*t*_14_=3.92, *P*=0.001). Also, there was a significant difference between two groups of control and aerobic training in the caspase-9 (*t*_14_=7.6, *P*=0.039) and caspase-3 (*t*_14_=11.5, *P*<0.001) in which the values of caspase-9 and 3 gene expression in the training group were more than the control group (Figure 3).

**Table 1:** Aerobic training protocol at introduction stage and eight-week training period

Repetition	Time (min)	Speed (m. min^-1^)	Training period
5	30	12-10	introduction stage
5	30	14	First two weeks
5	35	16	Second two weeks
5	40	18	Third two weeks
5	45	20	Fourth two weeks

**Table 2:** Nucleotide sequences of primers genes expression

R. Primer	F. primer	Gene
5' ATCAGCACCAAGCACGTCAT 3'	5' AAGTCAGCAACGTGGAAGGT 3'	HIF-1α
5' TCTGCTGTGCTGTAGGAAG3'	5'GGCTGCTGTAACGATGAAG3'	VEGF
5'TCCATCAGGGTATCCTCTCC 3'	5'GGAGGCAAGCATAAGACTGG3'	Cytochrom C
5' TCTCCATTGTCATCTCCAGTTGC3'	5' AGTGCTTTCCTGTGCTATCTCTTC3'	Apaf-1
5'GAGCCCACTGCTCCAGAATG3'	5'GTCAAGTTTGCCTACCCCCA3'	Caspase-9
5' GAGTCCACTGACTTGCTCCC3'	5' GAGCTTGGAACGGTACGCTAA3'	Caspase-3
5' GTGCAGGGTCCGAGGT3'‎	5'GCCCGCTAGCTTATCAGACTGATG3'‎	miR-21

**Table 3:** Bodyweight (g) of BALB/c mice in experimental and control groups

**Group ** **Week **	**Experimental **	**Control **
1	16.36±1.38	18.00±1.21
2	16.70±1.55	18.20±1.22
3	17.21±1.30	18.80±1.33
4	17.43±1.25	19.17±1.43
5	17.29±1.27	18.77±1.55
6	18.12±1.41	19.54±1.49
7	18.47±1.39	19.73±1.53
8	18.63±1.43	19.91±1.64

* Indicates a significant difference between the two groups

* Indicates a significant difference between the two groups

**Figure 4:**
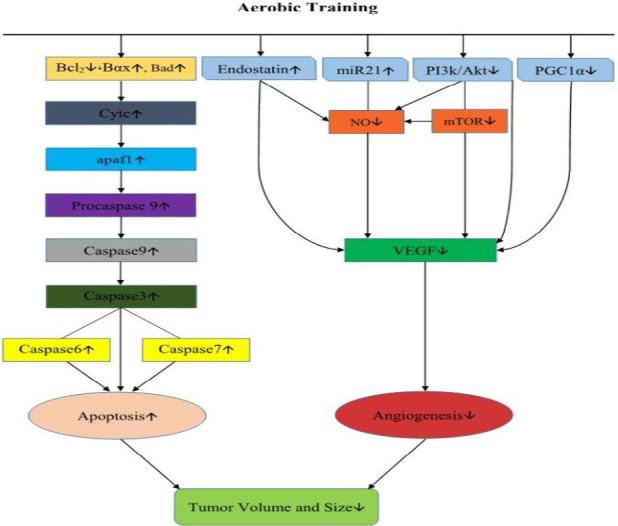
Aerobic training can reduce tumor volume and size by decreasing angiogenesis and increasing apoptotic factors

## Discussion

Despite advances in the treatment of many cancers, breast cancer is now one of the most common and deadly diseases in women (17). Therefore, the aim of the present study was to investigate the effect of 8 weeks of aerobic training on the gene expression of angiogenesis and apoptotic factors in mice with breast cancer. The results showed that eight weeks of aerobic training significantly reduced the tumor volume and gene expression of the miR-21. Also, eight weeks of aerobic training significantly increased the gene expression of HIF-1α, cytochrome C, Apaf-1, caspase-3, and caspase-9. However, VEGF gene expression did not significantly alter after eight weeks aerobic training.

The present study showed that aerobic training significantly reduced tumor volume compared to the control group, and the reduced tumor volume could be attributed to changes in the process of angiogenesis and apoptosis resulting from aerobic training. In our study, the aerobic training group had a significant increase in HIF-1α gene expression compared to the control group. Long-term aerobic exercises lead to increased hypoxia in patients, and in these conditions, a significant increase in HIF-1α gene expression and protein occurs. The HIF-1α complex, after formation, can detect hypoxia-reactive elements that lie on target genes (26).

Exercise is a strong physiological stimulus that deeply prompts mitochondria demands for O_2_ delivery of substrate to skeletal and heart muscles. This change in demand can indirectly conduct O_2_ substrate from the tumor to metabolically active tissues (for example, the heart and skeletal muscles). In this case, the direction for substrate access is activated by HIF-1α to maintain cell homeostasis (27, 28). Repeated endurance training can increase HIF-1α activity (27). Exercise increases the release of muscle-derived cytokines and increases the ligands associated with tyrosine kinase receptor attachment to the tumor and endothelial cells to stabilize HIF-1α expression, which occurs due to adaptation to exercise (29). HIF-1α is an important downstream target of miR-21 in regulating tumor angiogenesis (30). The reduction of miR-21 gene expression in tumor tissue has probably not been enough to reduce in HIF-1α expression. It has been demonstrated that growth factors, cytokines, and other signaling molecules can stimulate the synthesis of HIF-1α protein via activating phosphatidylinositol 3-kinase (PI3K)/AKT and mitogen-activated protein kinase (MAPK). Previous studies have shown that aerobic training can activate the mTOR pathway via the PI3K pathway, which in turn increases HIF-1α. In fact, mTOR is an upstream mediator of HIF-1α activation and PI3K/Akt signaling pathway could potentially regulate HIF-1α via mTOR (31). Also, it has been estabilshed that in hypoxia situations, interferon-stimulated gene factor 3 (ISGF3), signal transducer and activator of transcription (STAT3), and nuclear factor kappa B (Nf-_k_B) are attached to the promoter region of the HIF-1α gene to activate transcription initiation (32). Although HIF-1α is triggered by hypoxia and regular training (33) it seems that in this study, the intensity and duration of aerobic training have not been enough to control these important factors.

Our results also showed that aerobic training significantly reduced miR-21 gene expression and reduced VEGF gene expression in aerobic training. In line with our results, Wagner *et al.* reported that the baseline expression of miR-21 decreased after chronic exercise, independent of exercise levels, individuals with higher VO_2max_ had more reduced baseline gene expression than individuals with lower VO_2max _(28). The mechanism of the effect of exercise and especially aerobic training on miRs and their relationship with VEGF is not well defined. But, physical activities regulate miRNAs involved in cell proliferation, invasion, and metastasis. In addition, evidence has also shown that miRNAs can regulate the angiogenesis process by a direct effect on endothelial cells or through a direct effect on tumor cells (35). miR-21 is altered by resistance and endurance training and also participates in tumor invasion (36). In fact, laboratory studies have shown that in several types of cancer, miR-21 deficiency in mice revealed suppression of cell proliferation and tumor growth, and invasion and metastasis have also decreased (14). In addition, miR-21 negatively suppresses programmed cell death 4 (PDCD4) tumor and regulates the downstream signaling targets in the row of colorectal cells (34). Aerobic exercise can temporarily modify and reduce miR-21 expression (36). By reducing miR-21 expression within cancer cells, the exercise retrieves PDCD4 and phosphatase and tensin homolog (PTEN) activity and limits the proliferation of cancer cells. This pathway has been confirmed according to previous studies that have shown that exercise can regulate miR-21 in tissues or fluids, such as muscle tissue, in mice (37), as well as in plasma of healthy human subjects and patients with breast cancer (38). MiR-21 inhibition with aerobic training suppresses the signaling pathway. Inhibition of miR-21 also leads to a significant reduction in tumor growth and caspase activity (34). 

 Therefore, it is obvious that by reducing miR-21, tumor growth is reduced in the aerobic training group.

 Recent studies have shown that the HIF-1α/VEGF/VEGFR2 signaling pathway is activated by hypoxia. It is found that cross activity between HIF-1 and proangiogenic factors is a basic relationship during capillary formation under hypoxia. All steps of the vessel formation cascade are supported by HIF-1α and VEGF (39). Indeed this pathway is involved in endothelial cell proliferation, differentiation, migration, and vascular permeability (40). Since the expression of the HIF-1α gene has increased due to aerobic training, and this factor is one of the factors influencing the activation of VEGF gene expression, an increase in the expression of the VEGF gene in the tumor was expected, but its non-significant reduction in this study can be attributed to a significant reduction in miR-21 gene expression in this group. Isanejad *et al.* reviewed the effects of a combination of interval training and each of the two tamoxifen and letrozole on miR-21 and VEGF in mice with breast cancer. Their results showed a significant decrease and low regulation of miR-21 and VEGF (38). On the other hand, it has been documented that aerobic exercise can modulate VEGF by different signaling pathways included PGC-1α, ERR-α, and ERR-*γ* (38). PGC-1α is a master metabolic sensor that can regulate the expression levels of VEGF. Also, overexpression of ERR*γ* increased the expression of the VEGF gene. Another important factor that has a positive effect on VEGF expression in hypoxia situations is nitric oxide (NO) (41). On the other hand, Endostatin (ES) has been known as an antiangiogenic factor. It has been reported that ES prevents the elevation of VEGF by induction of apoptosis in endothelial cells, prevents the immigration of endothelial cells, and suppressing the signaling of NO (42). Although NO and ES gene expressions were not measured in the present study, it is possible that VEGF expression in the aerobic training group was decreased by reducing NO and increasing ES. Generally, in the present study, the intensity and duration of aerobic training were probably sufficient to inhibit the angiogenic factors in mice with breast cancer. However, this idea needs more studies in the future. 

According to the results of this study, aerobic training has increased the gene expression of cytochrome C, Apaf-1, caspase-3, and caspase-9 in tumor tissue. Because cancer is associated with uncontrolled cell growth with reduced apoptosis (43), the increase in the expression of apoptotic genes in the present study may have reduced the number of apoptotic cancer cells. According to some studies, hypoxia increased oxidative stress, and increased intracellular calcium ions can activate the internal pathway of apoptosis (44). Therefore, it is possible that aerobic training causes hypoxia (due to increased expression of HIF-1α gene in the present study) in cancerous cells, thus activating the gene expression of cytochrome C, Apaf-1, by activating the internal path of apoptosis. Caspase-3 and caspase-9 are increased. Another possible reason for the increase in the expression of apoptotic genes is a change in the production of oxidative stress due to aerobic training in the tumor tissue. Figure 4 has been summarized some mechanisms of the effect of aerobic training on angiogenesis and apoptotic factors in mice with breast cancer.

The process of reducing local blood flow in the tumor tissue and then re-establishing the circulation of tissue blood flow in this study can also produce reactive oxygen species (ROS) (45), which damages DNA by oxidizing purine and pyrimidine bases, especially guanine, and causes apoptosis of cancer cells (19). In addition to the association of miR-21 with angiogenesis in the tumor tissue, this factor makes cancer cells resistant to apoptosis, or say, suppresses apoptosis (46). In other words, miR-21 exhibits its oncogenic action by inhibiting cell apoptosis (47). Because miR-21 gene expression in the aerobic training group has decreased, its reduction may have led to an increase in apoptosis and apoptotic factors by releasing cytochrome C from the interstitial space into the mitochondria of cancer cells in the tumor tissue (46). Interaction of cytochrome C with ApaF-1 activates caspases 3 and 9, which ultimately increase or initiate apoptosis (45, 48). The present study had some limitations such as lack of evaluation ROS, Bcl-2, and Bax. Therefore, it is recommended to use the Western blot method in future studies. It is suggested that due to the inhibitory effect of Bcl-2 and stimulant effect of Bax in the apoptosis process and the role of ROS in angiogenesis and apoptosis process, in future studies, Bcl-2 and Bax gene expression and ROS levels should be measured to better evaluate the effect of aerobic training. It is also suggested to use the Western blotting method in future studies to determine the alteration in angiogenesis and apoptotic factors after aerobic training.

## Conclusion

The results of the present study show that regular aerobic training can play an effective role in reducing angiogenesis and increasing apoptosis activity in the breast tumor tissue, thereby reducing the growth rate of breast tumors in mice.

## Authors’ Contributions

MMR participated in most of the experiments including data collection, data analysis and writing the manuscript; RS participated in data collection, data analysis and writing the manuscript; MRK carried out the design of study, writing the manuscript and also supervised the project; RN carried out the design of study, contribution in data analysis, edited the manuscript, provided comments on the laboratory analysis and also was responsible for reviewers’ comments; AAG carried out the design of study and also supervised the project; All authors have read and approved the content of final manuscript.

## Conflicts of Interest

None declared. 
